# Detection of Dental Apical Lesions Using CNNs on Periapical Radiograph

**DOI:** 10.3390/s21217049

**Published:** 2021-10-24

**Authors:** Chun-Wei Li, Szu-Yin Lin, He-Sheng Chou, Tsung-Yi Chen, Yu-An Chen, Sheng-Yu Liu, Yu-Lin Liu, Chiung-An Chen, Yen-Cheng Huang, Shih-Lun Chen, Yi-Cheng Mao, Patricia Angela R. Abu, Wei-Yuan Chiang, Wen-Shen Lo

**Affiliations:** 1 Department of General Dentistry, Chang Gung Memorial Hospital, Taoyuan City 33305, Taiwan; harrythebold@cgmh.org.tw (C.-W.L.); mr2005@cgmh.org.tw (Y.-C.H.); louiszzzzz@cgmh.org.tw (Y.-C.M.); 2 Department of Computer Science and Information Engineering, National Ilan University, Yilan City 260, Taiwan; 3 Department of Electronic Engineering, Chung Yuan Christian University, Taoyuan City 32023, Taiwan; g10776018@cycu.edu.tw (H.-S.C.); g10976016@cycu.edu.tw (T.-Y.C.); s10726325@cycu.edu.tw (Y.-A.C.); s10726334@cycu.edu.tw (S.-Y.L.); s10726335@cycu.edu.tw (Y.-L.L.); Vincent_Lo@cycu.edu.tw (W.-S.L.); 4 Department of Electrical Engineering, Ming Chi University of Technology, New Taipei City 243303, Taiwan; 5 Center for Internet of Things and Intelligent Cloud, Chung Yuan Christian University, Taoyuan City 32023, Taiwan; 6 Department of Information Systems and Computer Science, Ateneo de Manila University, Quezon City 1108, Philippines; pabu@ateneo.edu; 7 National Synchrotron Radiation Research Center, Hsinchu City 30076, Taiwan; chiang.wy@nsrrc.org.tw

**Keywords:** biomedical image, periapical image, apical lesion, Gaussian high pass filter, iterative thresholding, deep learning, CNN

## Abstract

Apical lesions, the general term for chronic infectious diseases, are very common dental diseases in modern life, and are caused by various factors. The current prevailing endodontic treatment makes use of X-ray photography taken from patients where the lesion area is marked manually, which is therefore time consuming. Additionally, for some images the significant details might not be recognizable due to the different shooting angles or doses. To make the diagnosis process shorter and efficient, repetitive tasks should be performed automatically to allow the dentists to focus more on the technical and medical diagnosis, such as treatment, tooth cleaning, or medical communication. To realize the automatic diagnosis, this article proposes and establishes a lesion area analysis model based on convolutional neural networks (CNN). For establishing a standardized database for clinical application, the Institutional Review Board (IRB) with application number 202002030B0 has been approved with the database established by dentists who provided the practical clinical data. In this study, the image data is preprocessed by a Gaussian high-pass filter. Then, an iterative thresholding is applied to slice the X-ray image into several individual tooth sample images. The collection of individual tooth images that comprises the image database are used as input into the CNN migration learning model for training. Seventy percent (70%) of the image database is used for training and validating the model while the remaining 30% is used for testing and estimating the accuracy of the model. The practical diagnosis accuracy of the proposed CNN model is 92.5%. The proposed model successfully facilitated the automatic diagnosis of the apical lesion.

## 1. Introduction

**Figure 1:**
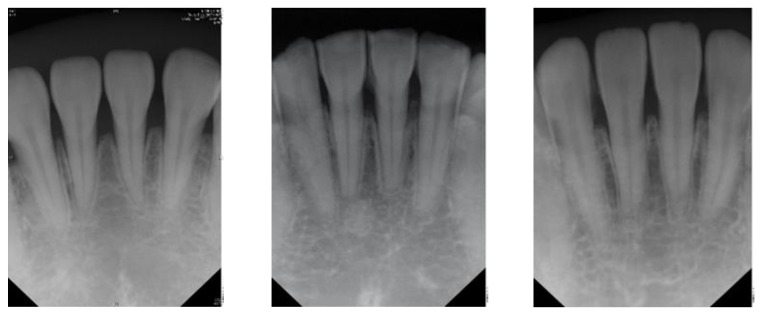
Periapical film database.

The radiographic finding of a potential endodontic pathosis is an essential part of endodontic assessment in daily dental practice. Apical periodontitis (AP) is an inflammatory response in the apical periodontium that often results from tooth root canal microorganisms, which may cause apical bone resorption, resulting in apical lesion [1]. Thus, diagnosis of periapical pathology has always been a topic of concern in endodontics. Identifying the periapical pathology of nonvital teeth is a key factor of successful treatment planning and prognosis evaluation [2]. Due to the fact that tooth with periapical pathosis is sometimes asymptomatic, it is often only detected via routine radiographic examination [3]. Few diagnostic tests provide as much useful information as dental radiography. In general, apical endodontic pathosis appears radiographically as bone loss in the area of the periapex and presenting radiolucent area at the apex of the root. Among all types of dental radiography, the periapical film which like Figure 1 is most commonly used in apical lesion diagnosis clinically [4].

Although cone-beam computed tomography(CBCT) is more sensitive in detecting apical lesions [5,6], the periapical film is still important, owing to the fact that periapical film requires lower radiation dosage exposure and is more commonly used in the daily dental radiographic examination [7]. Assessment of the location and extent of apical periodontitis (AP) will determine the treatment plan and subsequent treatment modality [8]. Therefore, it is necessary to be very cautious about the accuracy of the differential diagnosis of AP and normal periodontium, because each judgment has a huge impact on the treatment course of the patient [9]. However, the radiographic appearance of endodontic pathosis in a periapical film is sometimes highly subjective. In a study by Goldman et al., there was only 50% agreement among interpreters for the radiographic presence of pathosis. When the cases were reevaluated several months later, the same evaluators agreed with their own original diagnosis less than 85% of the time [10]. This also shows that in image interpretation, dentists make different judgments on the same image from time to time. Additionally, diagnosis of potential endodontic pathosis may be time-consuming for dentists in daily dental practice. With the rapid growth of technology, big data analytics, and machine learning techniques have been widely used in medicine, such as in autism [11], detection of pneumonia [12], risk of falls [13], classification of cancers [14], application of disease detection [15], predicting severe retinopathy of prematurity [16], dynamic modeling of medical imaging [17], and lung and pancreatic tumor characterization [18]. In response to this problem, this article combines big data analysis and machine learning technology to improve. Apical lesion detection by using periapical radiograph based on transfer learning with convolutional neural networks (CNN) can provide objective radiography interpretation and could potentially save more clinical time for dentists allowing them to focus on treatment modality and clinical operation.

Krajnc et al. [19] provided one of breast cancer detection by using machine learning the improvement took advantage of the advanced data pre-processing techniques in radioactive models. The differentiation was added for benign and malignant tumors in patients’ patterns that cannot be assessed sufficiently with conventional breast imaging and who are not candidates for MRI. Thus, the concept of the article is to develop a new technology to improve detection accuracy. The literature in [20] applied image preprocessing methods which included image resizing, center cropping, and intensity normalization solving the issues of dominant prostate pixels by inter-patient, inter-scanner variability, and background pixels. In order, to enrich the network with more data, incrementing variable data, accuracy improvement, patch extraction, and data enhancement are applied before network training.

Most of the studies are based on the analysis of oral X-rays for finding the characteristics of the disease. The methods for achieving the purpose of identifying the medical condition are directed toward the use of artificial intelligence. One is the example of the usage of artificial intelligence [21] that develop a new CNN model to determine the condition of the three dental caries of the panoramic image. The overall accuracy [22] is up to 86% using CNN for detecting teeth and classifying problems. Semantic segmentation was first used to distinguish between targets of interest with an accuracy of up to 89%. Other technologies do not use artificial intelligence [23] in determining the feature of the tooth decay of the bitewing image, but use edge identification and Otsu thresholding for the image preprocessing. The affected areas were highlighted by the connected component and mask to find out where dental caries are [24] and analyze panoramic images on the mandibular bone for osteoporosis detection. The GLCM matrix is used to find the features. Then, the SVM algorithm is used to classify normal and osteoporosis classes. The classification results are validated by using data prepared by the Dental Radiographs Department. In this study, the method for analyzing the image of the periapical and prejudging whether the apical is inflamed and developed has improved to an accuracy of 85.71%. A semi-automatic method is presented in the study in [25]; where the lesions were manually selected using the TopHat technology in calculating and differentiating between the features of the symptoms and the normal conditions. The deep-learning techniques are currently being wildly used. The article in [26] provided a method to enhance feature extraction including shape, color, and texture by applying a convolutional neural networks (CNN) architecture to extract deep-learning functions. Moreover, the function is a pre-trained ImageNet (ILSVRC ImageNet task). The proposed method in the literature is different from other works that choose the CNN as a technique to detect the apical lesion of the periapical images. The CNN automatically extracts the significant features and improved the accuracy by classifying the images for the proposed model. The semi-automatic method utilized logic regression analysis and provided an improved accuracy of 80.7% [25] for detecting the tooth decay and lesion of the periapical image. The active contour method and level set method were used to cut out the targets of interest while FVM was used to extract the features. It is also suggested that the accuracy can be improved by using fuzzy clustering [27] for detecting the granuloma of the periapical image. Gabor wavelengths were used to extract features from a periapical image, and SVM was adopted as a classifier. The reported accuracy reached as high as 91.67%. Based on the above information, this article proposes apical lesion detection by using a periapical radiograph based on transfer learning with convolutional neural networks (CNN), using the literature in [23,27] as important references for comparison. This provides objective radiography interpretation and potentially saves more clinical time for dentists, allowing them to focus on treatment modality and clinical operation.

## 2. Materials and Methods

**Figure 2:**
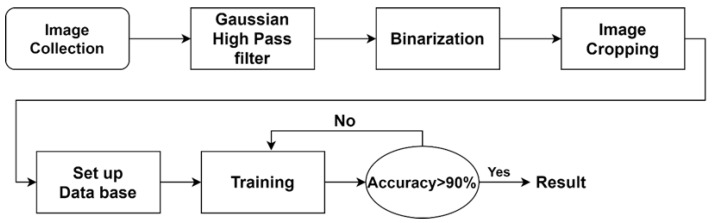
Proposal flowchart.

The purpose of this study is to identify the apical lesions of the periapical image through convolutional neural networks. The proposed approach presented in this study shows in Figure 2, can be divided into four steps: image processing, image cropping, retouching the setup database, and CNN image identification.

### 2.1. Image Preprocessing

**Figure 3:**
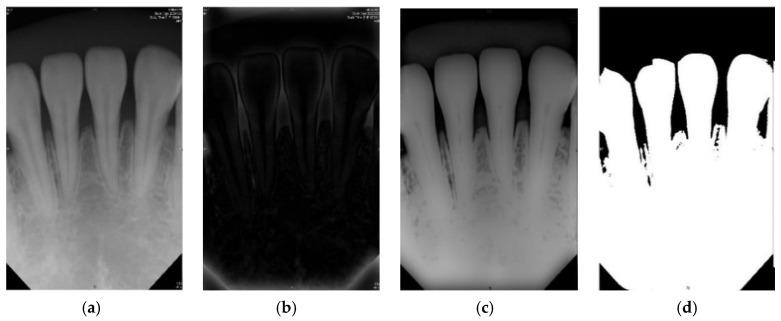
The preprocessed image results of this article. (**a**) Periapical film; (**b**) filtered result of the Gaussian high pass filter; (**c**) difference of (**a**) and (**b**); (**d**) result after the binarization.

The purpose of the image preprocessing step is to perform the binary processing [28] on the root tip. The advantage of binary processing is to effectively emphasize details that are difficult to find in the image or to separate the region of interest from non-targets. The pen tip is binary, and its background is changed. It is a very small gray value (i.e., 0). The bones of the teeth and grooves become the maximum ash value (i.e., 255). This step is performed by the subsequent imaging and cutting techniques [29]. According to the above references, if the original dental X-ray image is directly selected, such as using a bitewing image, and directly perform iterative threshold and binarization (two-value), the result will not be as expected. However, in the original clinical images (background, teeth, and grooved bones), the grayscale values of each segment may not be significantly different. It is not necessarily as distinct as described above, each segment is obvious and does not interfere with each other. The root tip is no exception, and there may be uneven grayscale values. In this case, it cannot be considered as the target and the background is indeed separated. With that, there will be incomplete dental images. If this problem occurs, it will seriously affect the subsequent image cutting. Figure 3 shows the results of image preprocessing in this paper.

#### 2.1.1. Gaussian High Pass Filter

Due to the problems mentioned in the other methods proposed in the literature, this article takes advantage of the Gauss Qualcomm filter first, followed by using the iterative algorithm to select the threshold to avoid the problems encountered. The Gauss high pass filter is explored, which enables the sharpening of the image and better extracts the edge information in the image. The formula in Equation (1) is used, which makes use of an image with size u×v:

(1) $$H\left(u,v\right)=1-e^{\frac{-D{\left(u,v\right)}^{2}}{2\times D_{0}}}$$

In this study, where the root tip by filter sharpening the image is performed, its filtered image shows the highlighted impurities and edges. The filtered image [29] is then subtracted to the original image. By doing so, the original image with impurities is reduced and the gray-scale value difference is more evident.

#### 2.1.2. Iterative Thresholding

There are many ways to choose the threshold value. The threshold value can either be a fixed threshold value or a variable one where an adaptive threshold value is implemented through many different ways of selection. The adaptive threshold is the main technology because it is based on a given image to calculate the best threshold so as to obtain a better two-value results. On the other hand, as using a fixed threshold is sensitive to noise, the differences among the gray-scale values become less obvious. The image segmentation with highly overlapping area among different target gray-scale values make the region of interests less evident. For that reason, the said method is seldom used.

In the case of using an adaptive threshold, the principle of the iterative threshold is to define the initial threshold, T0, as the average grayscale value. The practice is as follows (a)~(c):

(a) Finding the maximum (Z_max0) and minimum(Z_min0) grayscale value of the image to get the initial threshold value(T0).
(b) Using T0 as the cutting condition divide the image into two grayscale group and find the average grayscale value of two group (Z_out_ and Z_back_).
(c) Calculating the average of the Z_out_ and Z_back_ to find the new the threshold value (T1).

Lastly is to compare the gap conditions for the computed T1 and T0, like Equations (2) and (3). If the conditions are all met, T1 is the best threshold value as determined by the iterative algorithm [30].

(2) $$T0=\frac{Zmax0+Z\_min0}{2}$$

(3) $$T1=\frac{Z\_out+Z\_back}{2}$$

### 2.2. Image Cropping and Retouching

The introduction of image-based diagnosis, machine learning methods in disease prognosis, and risk assessment in the biomedical field paved the way to the CNN model for symptom classification established in this paper. This is performed by inputting a photo of a tooth to the network, and then the model tries to assess whether the tooth being examined has any symptoms or none. To improve the accuracy of judging the symptoms for each tooth in the periapical film (PA), it is separated into individual tooth images and the photos are preprocessed for noise removal. To avoid miscalculations of the CNN model, it is important to eliminate the noise content of the teeth in the non-primary judgment and the unnecessary portion of the trimmed image.

The previous papers related to image segmentation focused on atlas-based segmentation, model-based segmentation, and deep learning-based segmentation. Moreover, automatic image [27] focuses on prostate segmentation based on deformable models, region-based, and patch-based CNNs. The center cropping and intensity normalization [19] were used to separate each tooth by finding a cutting line with its adjacent teeth in a two-valued PA. The identified cutting line is separated from each tooth in the PA into individual tooth photos. However, in this technology [22] cut photos with incomplete pieces results to noisy images. This problem can be resolved by cropping the photos, segmenting the target image, and trimming the finished cropped output photo. The goal of the trimming technology is to cover the non-target image. The image is compared with the original image, and the non-target image is completely covered, but leaving the target image only. Finally, the non-target noise image masking technology is realized, which simplifies the image.

#### 2.2.1. Introduction to Vertical Cutting

Vertical cutting requires the use of a vertical projection in the photo to find a cutting line that separates adjacent teeth, and the step is to add up the pixel values of each column in a binary image to find the column with the smallest gross value. The principle behind this is that a cutting line that separates adjacent teeth will inevitably be on the tooth seam, and the tooth seam will be black after the two-value, with a pixel value of 0. Therefore, the location of the cutting line will be in the column with the smallest sum of pixel values. However, the teeth in the original image are not necessarily at the same angle. Some images perform the cutting line searching, which will cause the error of cropping to the target image. Thus, before using the vertical projection to search for the cutting line, the photo rotation must be corrected first. With the cutting line, the input photo is cut into slices of specific sizes. The two output photos are called the left photo after cutting and the right photo after cutting, which are respectively the area on the left of the cutting line and the area on the right of the cutting line. In order for the two output photos to present complete targets and reduce noise, it is necessary to determine which areas of the input images the output image is in. Here, the target of the left photo after cutting is the area to the left of the cutting line in the input photo, and the target of the right photo after cutting is the area to the right of the cutting line in the input photo. Therefore, the area of the left photo after cutting must be from the left edge of the input photo to the right end of the cutting line, and the area of the right photo after cutting must be from the left end of the cutting line to the right edge of the input photo.

**Figure 4:**
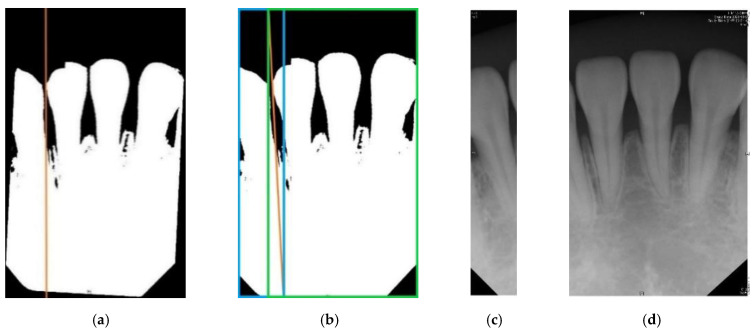
(**a**) Schematic diagram of the cutting line found by vertical projection; (**b**) schematic diagram of the cutting range; (**c**) and (**d**) are the output photos after vertical cutting the photo in (**b**).

Take Figure 4b as an example: the orange line in the figure is the cutting line found by vertical projection [31]. The blue box is the area of the left photo after cutting in the input photo, and the green box is the area of the right photo after cutting in the input photo. In order to separate each tooth in the input photo into photos of a single tooth after vertical cutting, it is necessary to judge whether the width of the output photo is less than 1/4 of the width of the input photo. If it does, the vertical cutting is put on halt. Otherwise, the vertical cutting step is repeated.

#### 2.2.2. Description of Image Retouching Execution Method

**Figure 5:**
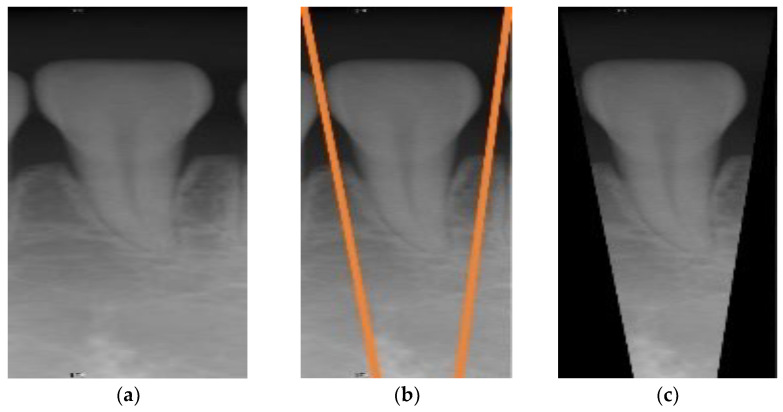
(**a**) The photo output after Image cropping, (**b**) the schematic diagram of Image retouching (the orange line is the cutting line, between the orange line is the target, and the rest of the area is the non-target), (**c**) the image after retouching.

In the vertical cutting, there will inevitably be noise images, that is, non-target images. In order to obtain more effective image data for improving accuracy, the output photos after image cropping must proceed to image retouching. The method is to set the pixel value of the non-target object to 0, and the area of the non-target object is outside of the two cutting lines, as shown in Figure 5. This part is done automatically.

### 2.3. Setup Database

**Figure 6:**
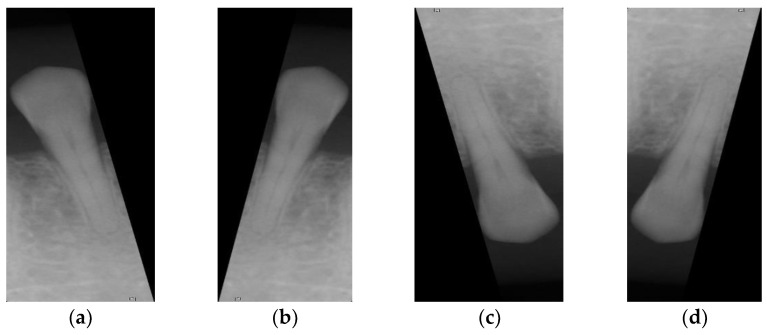
Image after standardization. (**a**) Original image, (**b**) horizontal flip, (**c**) vertical flip, (**d**) vertical and horizontal flip.

Clinical images were annotated by three professional dentists, having at least 4 years of clinical experience. The dentists guided the researchers, providing knowledge of the periapical radiographic finding and using actual cases to teach the researchers (describing the characteristics of apical lesions). They also provided the researchers clinical data to calibrate the CNN model (eliminating other nontarget symptoms). For the cut image, the first classification, a total of 191 root tip pieces were cut, afterward obtained 476 separate tooth images. According to the clinical database provided by the dentist, this paper distinguishes the images into normal and lesion. Due to the limited information provided, it is found that the samples in the database are unbalanced with a wide disparity between the number of normal and the number of the lesion, as shown in Table 1. If only a small number of images with apical lesions are used in CNN training, the CNN model will not be able to exert its advantages, so the learning effect is not good enough and the result cannot be judged correctly. To solve this problem, this article adds image augmentation technology during CNN training. According to the method of data enhancement [32], the following transformations are introduced: flip, zoom, rotation, translation, contrast, brightness, vertical flip, and horizontal flip, as shown in Figure 6. This technique increases the number of apical lesions to 131. At the same time, the input of normal images will be reduced during training. This converges the ratio between the two sets of samples, thereby reducing imbalance. The data enhancement step is only used to train the CNN model. Therefore, this did not cause confusion when verifying the CNN model.

The image after the normalizations step is shown in Figure 6a. Table 1 shows a six-time difference between the number of lesions and normal. With this amount of data put directly into CNN training, CNN will not be able to fully use its advantage and will not be effective in learning. Therefore, the usage of vertical flip and horizontal flip is increased, the number of lesion images is increased and made equal to the number of normal images, in this case 230 for both lesion images and normal images, giving a total of 460 images were built into the database. Allowing the proportions of the samples to be closer to equilibrium.

If the image cutting size is too large, the training process will take a longer time, causing network problems and resulting in low accuracy. So in this 460 image size standardization, the option is to use a resolution of 200 × 100. The time and network problems are resolved but resolution is traded off.

### 2.4. CNN Image Identification

Deep learning is a type of machine learning based on artificial neural networks, whose purpose is to train computers to perform human-like tasks. To simulate the way the human brain works, the system is expected to achieve the same learning ability as humans, including identification of objects, speech recognition, make decisions, or make predictions. In short, a large amount of regular information is provided to the computer, automatically finding the best function after the training process.

There are many deep learning networks in the scientific community such as convolutional neural networks (CNN), recurrent neural networks (RNN), and deep neural networks (DNN), with each of this network used in different specific applications. Considering this study, with the aim to ascertain the symptoms in the periapical image, CNN is the best method to apply. CNN is the most powerful method for image recognition, in dealing with convolutional layers to automatically fetch features and carry out feature integration and analysis.

#### 2.4.1. Adjust Model

Before CNN training, the model and database are needed. The database will be used in the step of image cropping and retouching, then the model is developed. Deep learning and neural network-like functions are used to describe data. With the objective function parameters well-defined, the input data is transformed into prediction results. In building-up the network architecture, a select group of possible features for the subsequent deep learning training process is first established. Proper network architecture is defined to generate an effective deep learning model through the training process. A pre-trained model has learned how to recognize the basic features of an image. For example, color, edge, curve, etc. On this basis, training time is reduced and problems that may be encountered while creating a new model from the beginning are avoided thereby improving the training efficiency. According to Yang et al. [31], its technical description of oral cancer in deep learning lies in the selection of adaptive modules as the goal of deep learning. This approach will be adapted in this study for the unique characteristics of the disease in the image to build automated module training. Overcoming the technical difficulties such as identification and marking results to a reduction of cost and improvement in the accuracy of deep learning. The concept of adaptive modules is used as a basis for adjustment and allow modules to have convenience without data restrictions. The adjustment module is no longer a challenge and allows for more flexible use of current data. This article places the input layer indented 200 × 100 × 3 and finds the association positively.

Within CNN, each layer is closely related. In the case of the model stride and convolutional kernel, the size does not adjust. The full-connection layer will need to have adjustments. Making changes in the input of the full connection layer will results to changes on the number of neurons for the entire model. Thus, it is necessary to carefully choose the size to be changed. Too many neurons do not only increase the model complexity and easy for it to overfit, it also makes the calculation time increase and less efficient. The class output layer was changed to 2 in order to see if there is a lesion or not. Table 2 lists the input and output between the layers after the modification.

#### 2.4.2. Adjust Hyperparameter

This paper adopts the SGD gradient drop method to find the best result. This is based on the principle of an iterative algorithm to find the minimum of the loss function. The calculated error of the result and updating the model to obtain high accuracy as well as the impact of learning rates is very large, as detailed in Table 3. In the training data, if a large amount of data is applied to the network, it will lead to a longer training time and memory limitations. In this case, the use of a non-convex function coupled with the neural network will provide a locally optimal solution. Therefore, the concept of mini-batch is used that treats the data by parts when training and does the training just once. This allows a speed up in the model convergence and improves accuracy. There are three hyperparameters that require special attention:

(1) Learning Rate: The size of the learning rate will determine whether the neural network could converge to the global minimum value and to get a higher accuracy. A too high learning rate causes the loss function to not converge or miss the global extreme of the gradient. On the other hand, a too low learning rate will lead to a slow network convergence or make the neural network converge to the local extreme value which is not the best solution.
(2) Max Epoch: An Epoch means that all training data is fully passed through the neural network once. One Epoch makes the training less accurate. An appropriate increase in the number of Epoch will result in a better accuracy but will in turn increase the training time.
(3) Mini BatchSize: BatchSize is the number of samples for a training session. A fewer number for the BatchSize causes the loss functions to be difficult to converge and harder to improve on the accuracy. Too large of a BatchSize will increase memory capacity and the performance of the model will also decline.

#### 2.4.3. Training

When training CNN, the overall data is divided into three general sets in order to conform to the actual situation. They are the training set, the validation set, and the test set that is not put in the learning training. Seventy percent (70%) randomly selected images from the database (322 images) are used as the training and validation set. The remaining 30% is used as the test set (138 images). The training set and the validation set are trained into the network at the same time. After waiting for the network to be trained, about 125 remaining images are put into it as a test set to verify the accuracy of the network and generate a confusion matrix and its truth table.

## 3. Results

The verification set is used and fed into the network to evaluate the accuracy of the model. The resulting prediction of the model versus the actual labels of the images are monitored in order to calculate the accuracy of the CNN network. Using Equation (4), the computed accuracy of the model is 92.75%. Using Equation (4), as derived from the results, the accuracy of detection of apical lesions based on the technique presented on this paper has been success-fully increased to 92.75%. This is in contrast to the study in [23] which reported an accuracy of 80.7%. In [23], TOPHAT technology was used to crop the periapical, and the model was determined by logistic regression analysis. Although this study showed a significant improvement. However, the possibility of clinical application is unlikely, because of its technical accuracy rate. To further improve on the performance of the model in terms of accuracy, recommendations for future improvements are presented in the next chapter in order to eventually meet the standards for actual use in clinical medicine.

(4) $$Accuracy=\frac{Correct\;predict\;images}{total\;images}\times 100\%$$

**Figure 7:**
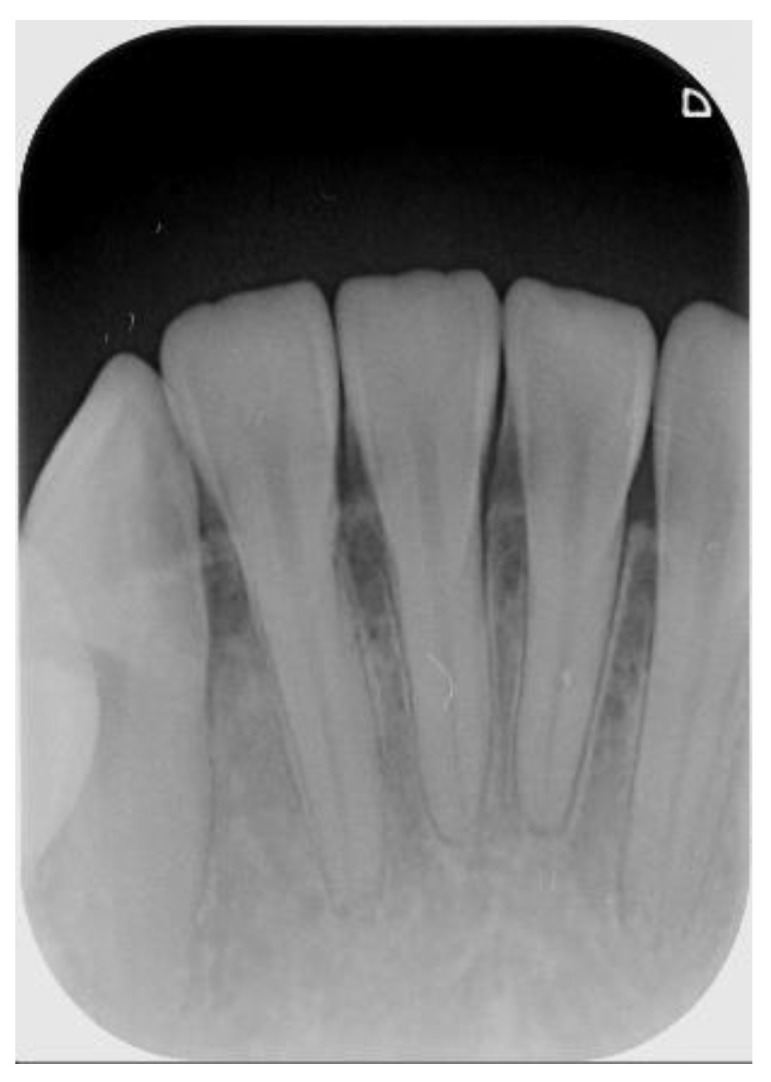
Outer example for validation (number 1 to 5 from left to right).

**Figure 8:**
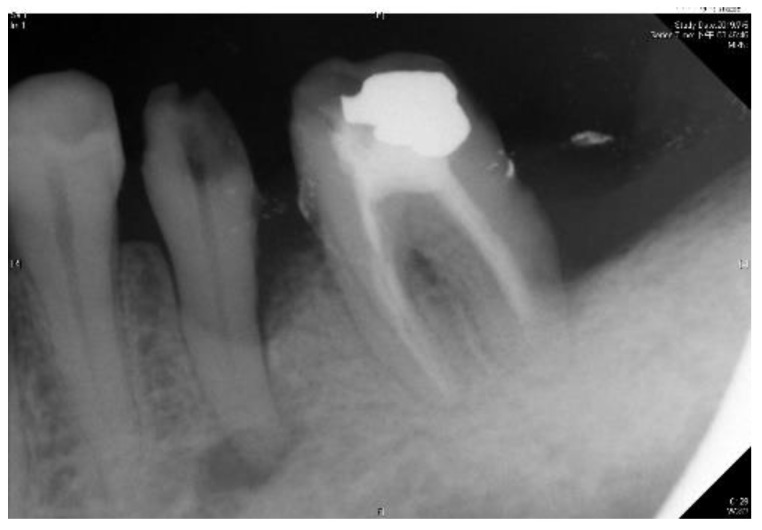
Outer example for validation (number 1 to 3 from left to right).

Table 4 shows that the larger the indicators the better is the accuracy. Table 4 also shows that the indicators in the proposed model are larger than that of the previous work in [23]. Consequently, the CNN image identification ability should be excellent especially in clinical medicine thus the need for high-precision judgment to provide better medical quality. The actual application of this technology uses the clinical images of Figure 7 and Figure 8 as the target image for judging the symptoms. After implementing this technology, the results are shown in Table 5 and Table 6.

**Figure 9:**
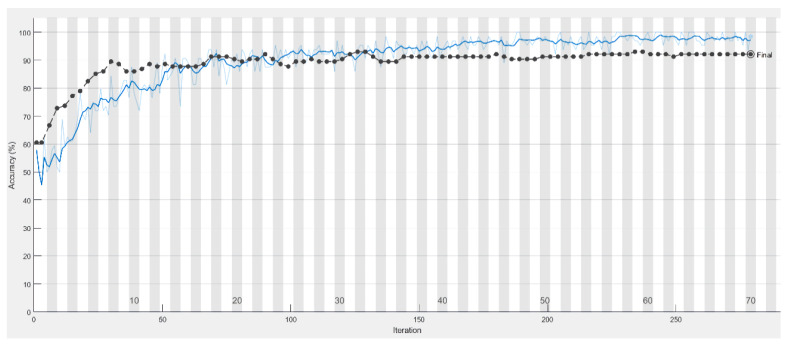
The training process of the model with the accuracy in the test set (black line) and the training set (blue line).

In this study, there are two main points to consider for accuracy improvement. For the first point, if a larger sized training input is fed into the network and the training result is not ideal, it implies that the loss function did not converge and that it took a long time for the training resulting in a low accuracy. With that, this article aimed to reduce the image size to solve the problem stated above. This is to remove the uninteresting and insignificant objects in the cut image to allow the CNN image recognition to focus more on the target of this article. Figure 9 shows that the proposed model and its accuracy rate contributed significantly, and its accuracy rate has grown significantly from 54.31% to 92.75% as listed in Table 7.

**Figure 10:**
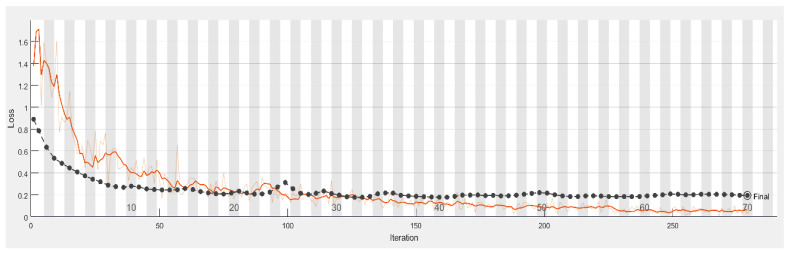
Loss process of the model with the accuracy in the test set (black line) and the training set (blue line).

It is shown from Figure 10 that the smaller the loss function, the higher is the accuracy of the reflection.

Table 8 and Table 9 present the resulting confusion matrix and accuracy for classifying apical lesion, respectively. From Table 9, the probability of misjudgment of being sick or not being judged to be sick has been controlled to 7%. The possible cause of this is an extremely mild disease and possible lesions of the apical tooth film image. The process of CNN image recognition may cause training confusion. It is expected that in the future, these subtle changes are learned and trained in a semi-supervised manner to reduce the probability of error judgment. Through the current image masking technology, the word order is improved to control the error rate. In Table 9, within 7%, in the future, it is also planned to optimize the characteristics of the symptoms by means of image processing. With that, the CNN can perform more accurately which determines the presence or absence of the disease with a view to higher accuracy.

To sum up, the accuracy of correct detection of apical lesions in this study has improved to 92.5%percent. For comparison, the paper by Orhan et al. with a fairly close topic is considered [33]. Orhan et al. (2020) investigated an evaluation of artificial intelligence for detecting periapical pathosis on cone-beam computed tomography scan. Its database consists of 153 images of root-tip-week lesions, and the CNN architecture was conducted using the U-net-like architecture. From the comparison table shown in Table 10, the accuracy of our model is quite close, and the accuracy compared with other papers is shown in Table 11. It can be seen that this model has a certain research value. In the future, we will continue to expand the database. Combining Fast R-CNN technology [34] to make the diagnosis of symptoms technology more accurate. And continue to strengthen the training volume of famous banks to achieve the goal of enhancing accuracy.

## 4. Discussion

The results of the current model in this work reached an accuracy of 92.75%. The outcomes indicated that there was a significant improvement compared with the literature [34], but there was still an improvement space in clinical medicine and the possibility of its future development. This scheme is designed to improve accuracy continuously and develop the practical application gradually of the medical system. For example, the PICCOLO data set [35] is used to build a powerful database to make the current overall performance more eye-catching, and the image reconstruction technology [36] is provided a good image reconstruction technology that uses image parameters adjustment to obtain a high SNR. The output of model results is optimized and automatically compared with the data provided by dentists. Convolutional neural networks can provide objective radiography interpretation and could save more clinical time for dentists to focus on treatment modality and clinical operation. There is considerable confidence toward future application development, which will be patented for these proposed results. Because this program is valuable and is a long-term continuous study, both the technology research and development results is to be protected and granted patents to enable more people to study and maintain the field of medical diseases.

Regarding the research limitations in this study, as it is impossible to collect all different dental apical lesions image data, the periapical radiograph image data sources collected in this study only comes from patients in Chang Gung Memorial Hospital. In addition, in terms of detection model selection, the CNN models (AlexNet, GoogleNet, Vgg, ResNet) were used in this study in consideration of both machine performance and accuracy. In the future, more deep learning models and more various periapical radiograph image data can be used. Although the CNNs can achieve excellent image recognition and detection results, it requires many data from various sources behind it and must be labeled as learning features.

## 5. Conclusions

Along with the image cropping process, it was found out in this study that cropping each tooth individually from a given X-ray image and trying to avoid ruining adjacent tooth while cropping them individually relies heavily on the ability to pre-process the images. Due to adopting the method of using the vertical sum of a pixel for cropping images, thereby binarization process, took an important role in the pre-processing step. Thus, taking advantage of the Gaussian high pass filter which has a critical capability to edge crispening led to improving the method of cropping that was implemented in this study.

On the other hand, to further improve on the success rate, two hypotheses are proposed. The first one is more intuitive, when the image data is increased it will be helpful to the improvement of the success rate. Secondly, is to enhance the characteristics of the Periapical lesions. Before using the image for CNN training, the area of the lesion is to be processed. As it is difficult to identify special cases for the CNN, this will allow the CNN to obtain new judgment data. The accuracy of this model represents the objective data for CNN to determine the lesions of Periapical lesions. The results presented in this study show the possibility of automatically identifying and judging the periapical lesions with a success rate of as high as 92.75%. This study paved the way in presenting an alternative way of automating recognition of apical lesions on periapical radiograph that are still done manually until the present.

## Figures and Tables

**Table 1:** Clinical application of the periapical image of the number of apical lesions.

Periapical Image of the Number of Apical Lesions			
	Normal	Lesion	Total
Quantity	411	65	476

**Table 2:** Input and output of each layer for the proposed CNN model.

Input and Output		
	Name	Feature Size
1	Input	200 × 100 × 3
2	Convolution	50 × 25 × 48
3	Relu	50 × 25 × 48
4	Normalization	50 × 25 × 48
5	Maxpooling	25 × 13 × 48
6	Convolution	13 × 7 × 48
7	Relu	13 × 7 × 48
8	Normalization	13 × 7 × 48
9	Maxpooling	13 × 7 × 48
10	Fully-Connected	1 × 1 × 2184
11	Relu	1 × 1 × 2184
12	Dropout	1 × 1 × 2184
13	Fully-Connected	1 × 1 × 2184
14	Relu	1 × 1 × 2184
15	Dropout	1 × 1 × 2184
16	Fully-Connected	1 × 1 × 2
17	Softmax	1 × 1 × 2
18	Class-output	2

**Table 3:** The hyperparameter values used in the proposed algorithm.

Hyperparameters	
Momentum	0.9
Initial Learning Rate	6 × 10^−5^
L2 Regularization	1 × 10^−4^
Gradient Threshold Method	l2norm
Gradient Threshold	Inf
Max Epochs	100
Mini BatchSize	128

**Table 4:** Accuracy (%) comparison.

Various Indicators Compared with [23]		
	Use Semi-Automatic Method Utilizing Logic Regression Analysis [23]	Test Accuracy
Accuracy	80.70%	92.75%
Sensitivity	80.00%	94.87%
Specificity	81.39%	90.00%
Precision	80.00%	92.50%
Recall	80.00%	94.87%

**Table 5:** Result after judgement for Figure 7 sample image.

Figure 7 Number	Clinical Data	This Study
1	Normal	99.6% to be normal
2	Normal	99.8% to be normal
3	Normal	98.6% to be normal
4	Normal	99.9% to be normal
5	Normal	99.9% to be normal

**Table 6:** Result after judgement for Figure 8 sample image.

Figure 8 Number	Clinical Data	This Study
1	Normal	98.5% to be normal
2	Apical lesion	99.5% to be apical lesion
3	Normal	97.3% to be normal

**Table 7:** Verbose.

Training Process						
Epoch	Iteration	Time Elapsed	Mini-Batch Accuracy	Test Accuracy	Mini-Batch Loss	Validation Loss
1	1	00:00:01	51.56%	54.31%	1.0615	0.7952
10	20	00:00:06	57.03%	71.55%	0.8295	0.5364
20	40	00:00:10	65.63%	81.03%	0.6642	0.4165
30	60	00:00:16	71.09%	87.93%	0.4652	0.3354
40	80	00:00:21	77.34%	91.38%	0.4305	0.2998
50	100	00:00:26	81.25%	92.24%	0.4062	0.2727
60	120	00:00:31	79.69%	92.24%	0.4079	0.2558
70	140	00:00:37	88.28%	92.75%	0.3160	0.2413
80	160	00:00:42	79.69%	92.75%	0.4439	0.2370
90	180	00:00:47	85.94%	92.24%	0.2881	0.2248
100	200	00:00:53	89.06%	92.75%	0.2672	0.2200

**Table 8:** The confusion matrix of accuracy.

The Confusion Matrix of Accuracy			
True Class	**Predict Class**		
		Healthy	Unhealthy
	Healthy	74 53.62%	4 2.89%
	Unhealthy	6 4.36%	54 39.13%

**Table 9:** Truth table of accuracy for classifying apical lesion.

Accuracy of Classifying Apical Lesion		
Actual Predicted	TRUE	FALSE
TRUE	92.50%	7.50%
FALSE	6.90%	93.10%

**Table 10:** Network comparison for apical lesion.

Network Comparison for Apical Lesion				
	AlexNet	GoogleNet	Vgg19	ResNet50
Accuracy	92.91%	89.36%	87.94%	88.65%
MaxEpoch	100	100	100	100
MiniBatchSize	64	64	64	64
Iterations per epoch	5	5	5	5
Max iterations	500	500	500	500
Validation patience	10	10	10	10
Learning rate	0.00006	0.00006	0.00006	0.00006
Elapsed time	1 min 15 s	3 min 53 s	81 min 8 s	167 min 32 s

**Table 11:** Comparison of accuracy with other papers.

Comparison of Accuracy with Other Papers						
	Our Method with Four Different Models of Transfer Learning				Method in [23]	Method in [27]
	AlexNet	GoogleNet	Vgg19	ResNet50	CNN	Fuzzy clustering and SVM
Accuracy	92.91%	89.36%	87.94%	88.65%	80.70%	91.67%
